# Pre-operative serum TSH levels: A risk factor for advanced metastatic differentiated thyroid carcinoma

**DOI:** 10.12669/pjms.35.5.704

**Published:** 2019

**Authors:** Sumera Batool, Muhammad Shakir Afridi, Adeel Khoja, Najmul Islam

**Affiliations:** 1 Dr. Sumera Batool, FCPS. Department of Endocrinology, Agha Khan University Hospital, Karachi, Pakistan; 2 Dr. Muhammad Shakir Afridi, Final year student MBBS, Agha Khan University Hospital, Karachi, Pakistan; 3 Dr. Adeel Khoja, MBBS, MSc. Department of Endocrinology, Agha Khan University Hospital, Karachi, Pakistan; 4 Dr. Najmul Islam, FRCP. Department of Endocrinology, Agha Khan University Hospital, Karachi, Pakistan

**Keywords:** Thyroid cancer, Differentiated thyroid cancer (DTC), TSH levels, American Joint Committee on Cancer (AJCC), Fine needle aspiration cytology (FNAC)

## Abstract

**Background and Objective:**

As the thyroid cancer incidence is increasing, the search for its risk factor is becoming more important. Serum thyroid stimulating hormone (TSH) levels being a growth factor for normal thyroid tissue, is also considered as growth promotor of cancer cells. In our study we aimed for pre-operative serum TSH levels of Differentiated thyroid cancers (DTC) done before their first surgery and determined its association with advanced disease in terms of stage, multifocal disease, lymph node involvement and distant metastasis.

**Methods:**

We have conducted a retrospective review of thyroid cancers from 1^st^ January 2008 to 31^st^ December 2017. Out of 281, 142 cases were included according to inclusion criteria. We noted the demographic details of participants, their histopathological diagnosis and serum TSH levels done before first surgery from the medical records. We calculated the stage of tumor through modified American Joint Committee (AJCC) staging system.

**Results:**

Out of 147 participants, 89.4% had papillary carcinoma or its variants whereas 10.6% reported follicular carcinoma. The mean pre-op TSH level of the patients included was 2.04 ± 1.79. In addition to the descriptive analysis, the univariate regression analysis revealed that the association of serum TSH levels was found to be statistically insignificant with advanced stage of thyroid cancer, multifocal disease, lymph node metastasis and distant metastasis respectively.

**Conclusion:**

The serum TSH levels before surgery was not associated with poor prognosis of differentiated thyroid cancer with respect to higher staging, multifocal disease, lymphatic or distant metastasis.

## INTRODUCTION

Thyroid cancer is the most common endocrine malignancy. Its incidence is rising and it has been estimated that it may become the 4^th^ common cancer in US by 2030. In Pakistan it accounts for 1.2% of all cancers.^1^ Both papillary and follicular carcinoma of thyroid are collectively called as differentiated thyroid cancer (DTC).^2^ Almost 90% of thyroid cancers are differentiated and among these, most common is papillary thyroid carcinoma (PTC) (70-80%) while 14% is follicular thyroid cancer (FTC).^2^

Increasing incidence of thyroid cancer can be attributed to better technologies available for diagnosis especially the common use of neck ultrasound to pick up small thyroid nodules and easy access to Fine needle aspiration cytology (FNAC). This is also supported by the evidence that developed countries have more number of thyroid cancer patients recently than the under-developed ones.^1^ Multiple factors affect the prognosis of DTC like age, tumor size and extrathyroidal extension, tumor stage and presence of distant metastasis.^2^

Thyroid stimulating hormone (TSH) act as a growth factor for thyroid nodules and responsible for thyroid hormone synthesis and release. The thyroid cells have TSH receptors and the same receptors are also expressed by thyroid cancer cells. Thus, TSH through these receptors might be responsible for growth of thyroid nodule and its possible conversion to thyroid cancer.^3^ Its suppression with the use of Levothyroxine is proposed to reduce the size of thyroid nodules and multinodular goiter. However, this approach is not recommended in current practiced guidelines and multiple studies have showed conflicting results.^4^ Based on this, serum TSH levels even within the upper normal range has been recently studied as a risk factor for thyroid cancer and its adverse outcomes.^3^ A metanalysis reported an association of high TSH with the high risk of thyroid cancer but on the other hand another study showed no such effect.^5^

The DTC prognosis is better if disease is treated at proper time. Even in patients operated for DTC, the chances of recurrence either local or regional, are 5-20%.^6^ So, the evaluation of risk factors responsible for DTC, is important so that they can be targeted at an early stage and prognosis of disease is improved.

Thus, to determine TSH as a poor prognostic marker for DTC in our population we have planned this study. In our study we aimed for pre-operative serum TSH levels of DTC done before their first surgery and determined its association with advanced disease in terms of stage, multifocal disease, lymph node involvement and distant metastasis.

## METHODS

This study was conducted in Aga Khan University Hospital Karachi, Pakistan, a tertiary care hospital with 721 bed capacity. It is a retrospective study which included all thyroid cancer patients presented to endocrine clinic from 1^st^ January 2008 to 31^st^ December 2017. We reviewed the medical record files of 281 cases and included 142 patients of differentiated thyroid cancer (DTC) into the study whose serum TSH levels were available, done pre-operatively within three months before surgery. The patients excluded were either anaplastic carcinoma, medullary carcinoma of thyroid, thyroid lymphoma or DTC patients whose pre-operative TSH levels were not available in hospital record. We also excluded the patients who were taking levothyroxine or anti-thyroid medications before surgery because these medicines alter the TSH levels. Data was collected by medical student under the supervision of endocrine fellow and noted in a pre-defined questionnaire. It included the demographic profile of patients, the histopathological diagnosis and stage of thyroid cancer, type of surgery performed and serum TSH levels. TSH levels were measured in Advia Centaur (Siemens Diagnostics) using Chemiluminescence immunoassay technique and its normal range is 0.4-4.0 mIU/L, which is a standard practice in our settings. The lymphatic or distant metastasis was documented through ultrasound neck, CT scans and Iodine I^131^ scans. The stages of all DTC were calculated through modified AJCC staging system.^7^

Primary objective was to assess the mean TSH levels before the DTC patients underwent their 1^st^ surgery and secondary objective was to see its association with the stage of DTC, multifocal disease and metastasis to lymph node and distant body parts.

### Ethical Consideration

We got the approval from the Institutional Ethical Review Committee (ERC number: 2018-0190-122). Throughout the study, we followed the core ethical principles of research. We coded each patient with a study ID and removed their name or hospital medical record numbers to maintain confidentiality. Moreover, the principles of justice, beneficence, confidentiality and non-maleficence were followed and maintained throughout the study.

### Plan of Analysis

Frequencies with percentages were reported for all independent categorical variables including the demographics and clinical variables as shown in Table-I. Mean with standard deviation (SD) was reported for continuous independent variables since the normality assumption was met as evident in Table-I.

**Table I:** Tabulated results of thyroid carcinoma patients.

S. No	Variables	n = 142 (%)
1.	Age*	41.03 + 15.9
2.	***Gender***	
1.	(a)Female	77 (54.2%)
1.	(b)Male	65 (45.8%)
3.	***Histopathological diagnosis***	
1.	(a)Papillary CA thyroid	127 (89.4%)
1.	(b)Follicular CA thyroid	15 (10.6%)
4.	Preoperative serum TSH levels*	2.04 + 1.79
5.	Ultrasound neck performed before surgery	94 (66.2%)
6.	Ultrasound guided FNAC performed before surgery	95 (66.9%)
7.	***Type of surgery performed***	
1.	(a)Total thyroidectomy	115 (81%)
1.	(b)Initial lobectomy followed by completion thyroidectomy	24 (16.9%)
1.	(c)Lobectomy alone	3 (2.1%)
8.	Neck dissection performed	74 (52.1%)
9.	***Stage of thyroid carcinoma***	
1.	(a)Stage I	103 (72.5%)
1.	(b)Stage II	31 (21.8%)
1.	(c)Stage IV	8 (5.6%)
10.	***Type of lesion on histopathology***	
1.	(a)Unifocal lesion	112 (78.9%)
1.	(b)Multifocal disease	30 (21.1%)
11.	Presence of lymph node metastasis	71 (50%)
12.	Presence of distant metastasis	22 (15.5%)

* Continuous variables with normality assumption met, mean with standard deviation is reported.

In order to gauge the association between serum TSH levels (dependent variable) and advanced stage of thyroid cancer, multifocal disease, lymph node metastasis and distant metastasis (independent variables), univariate linear regression model was utilized for statistical association. Table-II. P-value of <0.05 was considered statistically significant. All analysis was performed on Stata version 12.

**Table II:** Association of serum TSH levels (outcome variable) with advanced stage of thyroid cancer, multifocal disease, lymph node metastasis and distant metastasis (independent variables) - Linear Regression.

S.No	Variable	Beta Coefficient	Standard Error	95% C.I	*p*-value
1.	Multifocal disease (lesion type)	-0.42	0.37	-1.15 – 0.31	0.25
2.	Advanced stage of cancer (stage of the tumour) - Stage I was taken as reference				0.74
	Stage II	-1.15	0.37	-0.89 – 0.59	
	Stage IV	-0.44	0.63	-1.68 – 0.79	
3.	Lymph node metastasis	0.30	0.30	-0.29 – 0.90	0.32
4.	Distant metastasis	-0.17	0.42	-0.99 – 0.65	0.68

## RESULTS

Out of a total of 142 study participants, 127 patients (89.4%) had papillary carcinoma or its variant, while 15 (10.6%) had follicular carcinoma. Mean age of our study participants at the time of diagnosis was 41.03 ± 15.9 and it was more common in females 54.2% as compared to males 45.8%.

Mean TSH of study participants done within the last three months before surgery was 2.04 ± 1.79. Participants who had their ultrasound neck done before their surgery were 66.2% and FNAC was performed in 66.9% of participants for diagnostic purposes pre-operatively. After diagnosis 115 (81%) of the study participants underwent total thyroidectomy, lobectomy was performed in 3 (2.1%) participants and completion thyroidectomy, after initial lobectomy was done in 24 (16.9%) participants. Neck dissection was performed in 74 participants (52.1%), along with thyroidectomy.

With respect to histopathological diagnosis, 112 participants (78.9%) had unifocal lesion while 30 (21.1%) had multifocal lesion and mean size of the tumor was 3.7 cm. According to modified staging criteria, 103 participants (72.5%) were characterized as stage I, 30 (21.1%) as stage II, 8 (5.6%) as stage IV. There were no study participants in stage III. Lymph nodes were metastasized in 71 (50%) of the participants while distant metastasis was observed in 22 (15.5%) cases. The mean serum TSH level was 2.1, 2.0 and 1.3 in stage I, II and IV of DTC respectively.

In addition to the descriptive analysis, the univariate linear regression analysis revealed that the association of serum TSH levels (outcome variable) was found to be statistically insignificant with the independent variables; advanced stage of thyroid cancer (95% C.I’s: -0.89 – 0.59 for stage II &-1.68 – 0.79 for stage IV, p value=0.74), multifocal disease (95% C.I: -1.15 – 0.31, p value=0.25), lymph node metastasis (95% C.I: -0.29 – 0.90, p value=0.32) and distant metastasis (95% C.I: -0.99 – 0.65, p value=0.68).Descriptive Analysis is shown in Table-I and Inferential Analysis is shown in Table-II

## DISCUSSION

In our study, mean age of the study participants was 41.03 ± 15.9 years and it was found to be 1.2 times more common in females than males. Since 1960, ultrasound has been widely used for thyroid nodule evaluation and the American Thyroid Association (ATA) also recommends the assessment of lymph node involvement with neck ultrasound even if clinical examination is normal.^8^ In our institution, this guideline is followed in 66.2% of patients only and its use should be further emphasized for pre-operative staging. Similarly, ultrasound guided FNAC should be considered in thyroid nodules with suspicious features where it can provide accurate diagnosis in 91 to 93% of cases.^8^

In the past, total thyroidectomy was considered as the gold standard for Differentiated thyroid cancers (DTC) management but now ATA recommends that even lobectomy can be enough in carefully selected cases with low risk features like unifocal tumor of size < 4cm with no extra thyroid extension or lymph node involvement on clinical examination or imaging. In such patients, the survival rate is not different between lobectomy and total thyroidectomy.^9^ Similarly, neck dissection was part of initial surgery in 52.1% cases which is routinely not considered unless there is lymph node metastases clinically or radiologically.^10^ It seems we are still following a conservative approach with total thyroidectomy in 81% of participants and another 16.9% underwent two surgeries with initial lobectomy followed by total thyroidectomy. The prevalence of papillary carcinoma in our study is comparable to previous studies done in Pakistan and with the international data.^11^

According to modified American joint committee on cancer (AJCC) staging, 72.5% (n=103) of patients were characterized as stage I, 21.1% (n=30) as stage II, and 5.6% (n=08) as stage IV. In an Indian study, they found 68% of their patients as stage I and II while 31% of them were in stage III and IV collectively. Similarly, in another study the stages I and II had 85.35% patients while our patients of stages I and II combined were 93.6%. But the staging of thyroid cancer was modified recently and according to the modified AJCC classification, more thyroid tumors are being reclassified into low risk groups.^7^ This was applicable from 1^st^ January 2018 and we have used this modified version of staging in our study. This is the reason why our study is showing more patients in stage 1 and 2 as compared to other studies.

Similarly, lymph node involvement was found in 50% (n=71) and 15.5% (n=22) participants respectively had distant metastasis at the time of diagnosis in our study. This data differs from international studies which showed a histologically proven metastasis in lymph nodes in 69.6% (higher than our study) and distant metastatic deposits in 7.8% (much lower than our study).^12^ However, in a study in Egypt and another in Pakistan, the lymph node involvement was found in 40.9% of the study participants having PTC, results comparable to our patients.^13^,^14^ Similarly, distant metastasis incidence varies overall between 10 to 35% and previously reported incidence was 9% and 23% for PTC and FTC respectively in our area.^14^

In our study, we have determined the mean pre-operative TSH levels of all thyroid carcinoma patients enrolled and it was found to be 2.04 ± 1.79. Our mean TSH level is comparable with a previous study done in Pakistan where they found a mean TSH of 1.94.^15^ Similarly a study conducted in India showed, that majority of thyroid carcinoma patients (88.3%) had their TSH levels between 1.71 – 5.5 mIU/l and among south Korean population, it was found to be 1.95 ± 0.9 mIU/l.^3^,^16^ These results are comparable to our study as well.

However, when we compare the mean TSH levels between the DTC stages, it was found to be 2.1, 2.0 and 1.3 in stage I, II and IV respectively and there was no statistically significant association of higher TSH levels with the advanced stage of disease. Similarly, we did not find any association of TSH levels with lymph node involvement by DTC or distant metastasis and preoperative serum TSH levels was not found to be a risk factor for multifocal disease. These findings are in contrast to a study which showed a higher mean TSH in stage I/II than in stage III/IV i.e. 4.9 vs. 2.1 with statistical significance.^17^ These results were reciprocated in another study where TSH levels were high in stage T3/T4 and N1 than T1/T 2 and N0 respectively.^18^ But on the other hand, there are numerous studies which failed to show such association thus supporting our results. In analysis of 2184 DTC patients in South Korea, they found no relationship of TSH levels obtained before surgery with stage of thyroid carcinoma or tumor size.^3^ Subclinical hypothyroidism in patients of DTC was not found to be a risk factor for lymph node metastasis or multi focality of tumor as we have found in our patients.^19^ Similarly, another study found no association of TSH with distant metastasis like our study.^20^

Few studies have shown an association between TSH and advance stage of cancer i.e. high mean TSH in stage III/IV than in stage I/II in older age (>45 years) thyroid carcinoma patients.^19^ So, the difference in age group of participants studied might be a reason for different results in these studies. Moreover, the local data representing a link between thyroid malignancy risk and TSH has used different selection criteria and compared the characteristics of benign and malignant thyroid nodule. Our study included only the diagnosed DTCs and showed comparable mean TSH levels but does not indicate the role of TSH for advanced stage of DTC, multifocal disease, lymph node involvement or distant metastasis.

Thus, TSH might be responsible for growth of thyroid nodule in size but it is not associated with poor prognosis. So, it cannot be used as marker for pre-operative staging or characterization of patients into high risk group. Moreover, this study does not support that treating TSH levels of the patients with thyroid nodule even when they are within upper normal limit, would help to decrease the risk of thyroid cancer progression. We need to explore other options like molecular markers and should encourage the use of ultrasound neck and FNAC for the pre-operative staging of DTC which can reliable predict malignant characteristics of a thyroid nodule and lymph node involvement.

## CONCLUSION

Our study could not find any association of serum TSH levels with the thyroid carcinoma in terms of its advanced stage or metastasis. But our study has limitations as it is a retrospective study with a small sample size. Out of 281 cases we could only include 142 patients and the main reason for excluding half of patients was no follow up records as these patients were lost to follow up. The reason could be financial constrains or lack of awareness about the disease. In developing countries like Pakistan this poses a real challenge. DTC has a good prognosis if treatment is started as proper time with a proper follow up with endocrinologist. We need to educate and counsel our patients about the disease and its prognosis.
